# Impact of Deep Learning Denoising Algorithm on Diffusion Tensor Imaging of the Growth Plate on Different Spatial Resolutions

**DOI:** 10.3390/tomography10040039

**Published:** 2024-04-02

**Authors:** Laura Santos, Hao-Yun Hsu, Ronald R. Nelson, Brendan Sullivan, Jaemin Shin, Maggie Fung, Marc R. Lebel, Sachin Jambawalikar, Diego Jaramillo

**Affiliations:** 1 Radiology Department, Columbia University Irving Medical Center, New York, NY 10032, USA; 2 GE Healthcare, New York, NY 10032, USA

**Keywords:** diffusion tensor imaging, spatial resolution, denoising, pediatrics, growth, voxel size

## Abstract

To assess the impact of a deep learning (DL) denoising reconstruction algorithm applied to identical patient scans acquired with two different voxel dimensions, representing distinct spatial resolutions, this IRB-approved prospective study was conducted at a tertiary pediatric center in compliance with the Health Insurance Portability and Accountability Act. A General Electric Signa Premier unit (GE Medical Systems, Milwaukee, WI) was employed to acquire two DTI (diffusion tensor imaging) sequences of the left knee on each child at 3T: an in-plane 2.0 × 2.0 ${mm}^{2}$ with section thickness of 3.0 mm and a 2 mm^3^ isovolumetric voxel; neither had an intersection gap. For image acquisition, a multi-band DTI with a fat-suppressed single-shot spin-echo echo-planar sequence (20 non-collinear directions; b-values of 0 and 600 s/mm^2^) was utilized. The MR vendor-provided a commercially available DL model which was applied with 75% noise reduction settings to the same subject DTI sequences at different spatial resolutions. We compared DTI tract metrics from both DL-reconstructed scans and non-denoised scans for the femur and tibia at each spatial resolution. Differences were evaluated using Wilcoxon-signed ranked test and Bland–Altman plots. When comparing DL versus non-denoised diffusion metrics in femur and tibia using the 2 mm × 2 mm × 3 mm voxel dimension, there were no significant differences between tract count (*p* = 0.1, *p* = 0.14) tract volume (*p* = 0.1, *p* = 0.29) or tibial tract length (*p* = 0.16); femur tract length exhibited a significant difference (*p* < 0.01). All diffusion metrics (tract count, volume, length, and fractional anisotropy (FA)) derived from the DL-reconstructed scans, were significantly different from the non-denoised scan DTI metrics in both the femur and tibial physes using the 2 mm^3^ voxel size (*p* < 0.001). DL reconstruction resulted in a significant decrease in femorotibial FA for both voxel dimensions (*p* < 0.01). Leveraging denoising algorithms could address the drawbacks of lower signal-to-noise ratios (SNRs) associated with smaller voxel volumes and capitalize on their better spatial resolutions, allowing for more accurate quantification of diffusion metrics.

## 1. Introduction

Diffusion tensor imaging (DTI) can characterize tissue microstructure and microarchitecture inside a voxel of interest [1], thus providing new information previously unavailable from conventional magnetic resonance imaging (MRI). DTI techniques have been rigorously studied and well described within the fields of brain, spine, and nerve imaging [2,3,4,5,6]. The use of DTI in the physeal–metaphyseal complex for prediction of pediatric growth has been studied for approximately 10 years [7]. Characterization of columns of cartilage and newly formed bone in the physis and adjacent metaphysis through tractography has proven useful for the determination of height gain and the evaluation of growth failure in pediatric subjects [7,8,9,10,11,12,13]. Tractography is the result of tensor estimation inside each voxel; the tensor depicts the main direction of unrestricted water diffusion inside the columns running perpendicular to the growth plate [2,7]. 

Accurate quantitative DTI metrics rely on specific acquisition parameters and the achievement of satisfactory SNRs due to the intrinsic vulnerability of MR-DTI to artifacts caused by diffusion gradients and motion [1]. Ample studies have used DL to optimize DTI in the central nervous system, from basic denoising to using denoising algorithms to minimize the diffusion weighted data requirements, and even to asses sensitivity and specificity of the technique for classification of white matter disorders [14,15,16,17,18]. However, only a few studies have investigated the effects of varying acquisition parameters on DTI metrics in the orthopedic field, which were predominantly performed on adults or animal models, primarily focusing on adult muscle fibers or articular structures in rat knees [19,20,21,22,23,24]. These studies highlighted the sensitivity of knee connective tissues, specifically ligaments, to changes in spatial resolution [24]. Surprisingly, the rat knee physes demonstrated no significant variations in fractional anisotropy (FA) or mean diffusion across different spatial resolutions. Furthermore, the influence of these variations on physeal–metaphyseal tractographic diffusion metrics such as tract count, volume, and length remains unassessed [24].

Spatial resolution plays an essential role in ensuring the quality and reliability of DTI by influencing and modulating the occurrence of partial volume effects (PVEs) [25]. Larger voxel dimensions (associated with lower spatial resolution) offer higher SNRs but increase the probability of PVEs. In contrast, smaller voxel dimensions provide better spatial resolution and reduce the likelihood of PVEs at the cost of lower SNRs.

Our study aims to assess the impact of a deep learning (DL) denoising reconstruction algorithm applied to identical patient scans acquired with two different voxel dimensions, representing distinct spatial resolutions. We hypothesize that the denoising reconstruction algorithm will have a more pronounced effect on the smaller voxel dimensions, given their inherently lower SNR and consequent higher level of noise, which can be more effectively eliminated through the algorithm. Through this study, we hope to obtain valuable insights into the potential benefits of employing the denoising reconstruction technique in the context of varying spatial resolutions in DTI of the growth plate.

Our research presents a novel application of a deep learning (DL) denoising algorithm to DTI data of the knee growth plate. This application has not previously been explored or validated. The commercial software provided by the manufacturer was designed for denoising clinical structural images such as 2D T1 and T2 weighted scans. However, we will utilize a prototype denoising reconstruction algorithm applied to diffusion EPI scans, which is not yet commercially approved or tested for DTI scans. Our study will evaluate the effectiveness of this prototype denoising reconstruction in evaluating microstructural diffusion metrics, possibly demonstrating its potential to enhance the spatial resolution of DTI scans and offering novel insights into more reliable growth plate DTI metric changes over time. 

## 2. Materials and Methods

### 2.1. Subjects

A prospective study was conducted at our tertiary pediatric center, in compliance with the Health Insurance Portability and Accountability Act and approved by the institutional review board, to evaluate growth using DTI of the knee. Healthy girls (8–15 years old) and boys (10–16 years old) (14 girls, 13 boys) during the pubertal and adolescent expected growth spurt, were recruited between August 2022 and November 2023. Informed consent and assent were provided by every parent/legal guardian and child, respectively before they participated in the study. The study was conducted in accordance with the Declaration of Helsinki, and the protocol was approved by the Ethics Committee of Columbia University Irving Medical Center (AAAS9882).

### 2.2. MRI

We performed two DTI sequences of the left knee on each child at our pediatric center at 3T. We used a multi-band DTI acquisition with a fat-suppressed single-shot spin-echo echo-planar sequence (20 non-collinear directions; b-values of 0 and 600 s/mm^2^). Slice-selective gradient reversal was used for fat suppression. Two voxel dimensions were acquired on each subject, an in-plane 2.0 × 2.0 mm^2^ with section thickness of 3.0 mm and a 2 mm^3^ isovolumetric voxel, both without inter-section gap. We used a General Electric Signa Premier unit (GE HealthCare, Waukesha, WI, USA) with an 18-channel knee coil (Quality Electrodynamics, Mayfield Village, OH, USA). Parameters: repetition time (TR)/echo time (TE); 3000/51.7 ms; bandwidth 1953.12 Hz/pixel; parallel imaging factor, 2; signal averages, 5 for 600 b-value scans; matrix 128 × 128; field of view, 256 × 256 mm.

### 2.3. Intra-Voxel Tensor Visualization at Different Spatial Resolutions

To illustrate how acquisition at different spatial resolution (smaller versus larger 3D voxels) influences diffusion tensor direction, we employed MRtrix3 3.0.4 [26]. This software package is commonly used in diffusion imaging to visualize intravoxel tensors. The diffusion tensor is a mathematical model that characterizes the diffusion properties within a voxel, capturing the directionality and magnitude of water diffusion in three-dimensional space [27]. MRtrix3 uses the acquired MR-DTI data to estimate the diffusion orientation at each voxel [26], making it a useful tool to visualize and examine fiber tractography in the physes.

A diffusion-weighted image was selected as input for MRtrix3—2 mm × 2 mm × 3 mm volume. The volume was resampled into 2 mm^3^ image using MRtrix3’s regrid command. Confirmed successful resampling was achieved with the mrinfo command from MRtrix3 toolbox. Both the original and resampled images were saved into separate folders along with their corresponding .bval and .bvec files. For each corresponding image, the Dhollander algorithm was employed [28]. This method is instrumental in creating basis functions essential for estimating fiber orientation distributions (FODs) derived from the diffusion signal. Consequently, a model was established to project how the diffusion signal changes in different orientations and with varying diffusion gradients applied. The outputs from this algorithm provided the corresponding voxels used to build the basis function. Subsequently, the dwi2fod command from MRtrix3 toolbox was utilized to apply this basis function to each voxel in the input volume [29]. Finally, the mrcat command was used to concatenate these into a single volume, enabling the visualization of tensor ellipsoids that are representative of the fiber orientation directions.

### 2.4. AIRTM Recon DL Algorithm (GE Healthcare, Waukesha, WI)

The MR vendor provided a commercially available DL model with 75% noise reduction settings (Recon DL strength: High) applied. This model was applied on the same subject DTI sequences acquired at two different spatial resolutions (isovolumetric 2 mm^3^ and 2 mm × 2 mm × 3 mm).

### 2.5. Segmentation

Using fiber tract reconstruction software, Diffusion Toolkit v. 0.6.4 (trackvis.org, Martinos Center for Biomedical Imaging, Massachusetts General Hospital, Boston, MA, USA) and Trackvis (FACT algorithm), the brightest voxel inside the physes was used as the reference point to locate the physes. A region of interest (ROI) was drawn intersecting the distal femoral and proximal tibial growth plates perpendicular to the long axis of the bone on every slice. ROIs were manually drawn in the AIRTM Recon DL reconstructed scans (*n* = 54 DL reconstructed scans) over the distal femur and proximal tibia physes. The same ROIs were applied to non-denoised scans (*n* = 54 non-denoised scans) for consistency. Diffusion metrics (tract count, tract volume, tract length, and fractional anisotropy (FA)) were obtained from the resultant tractography. 

### 2.6. Signal-to-Noise Ratio Measurements

The signal-to-noise ratio (SNR) serves as an important metric when assessing the quality of DTI data. Enhanced SNR results in more dependable tensor estimation, consequently boosting the reliability and clarity of DTI-derived metrics, like FA, mean diffusivity, tract length etc. In MRI data, particularly DTI, noise can vary spatially due to elements like multi-channel coil sensitivity profiles, parallel imaging, and susceptibility artifacts. Traditional methodologies might not effectively capture this noise variance. To tackle this, SNR was calculated using the validated single-image set method applied by Wang et al. for the assessment of SNR in muscle diffusion tensor imaging [30]. Imaged volumes were paired with others that had proximally aligned diffusion encoding directions. We performed a subtraction of each DWI to yield initial noise image volumes. Each slice was transformed to k-space using 2D Fourier transformation, followed by Butterworth filtering and 2-D inverse transformation to image space, resulting in the final noise image volumes. Finally, the average noise variance was calculated from the same local ROI for both b0 and b600 images.

### 2.7. Statistical Analysis

We compared the tract count, volume, length and FA, derived from both reconstructed scans and non-denoised scans for femur and tibia, at each spatial resolution. Differences between spatial resolutions were evaluated using Wilcoxon-signed ranked test and Bland–Altman plots. All statistical analysis was performed on JMP^®^, Version <17>. SAS Institute Inc., Cary, NC, USA, 1989–2023.

## 3. Results

**Figure 1:**
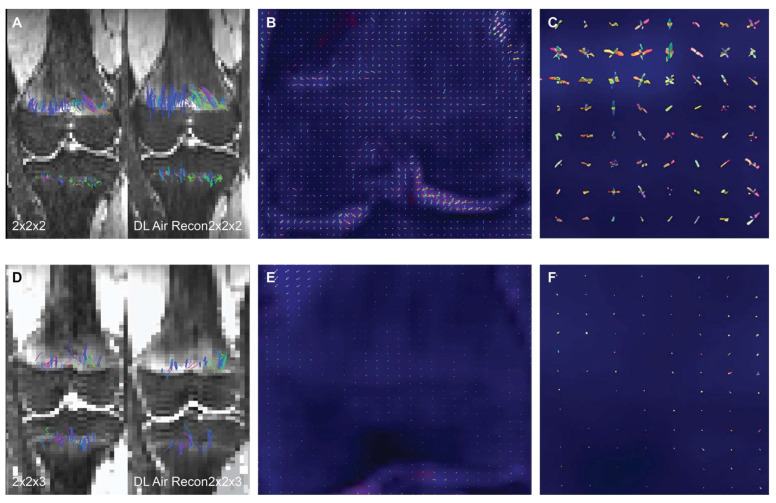
Same subject DTI acquisition using voxel sizes of (**A**) 2 mm^3^ and (**D**) 2 mm × 2 mm × 3 mm. The image quality is visibly better when DL reconstruction is applied in the 2 mm^3^ voxel size (**A**), with sharper bone contours and an increase in fiber tracts on both the femur and tibia. Conversely, in the 2 mm × 2 mm × 3 mm voxel size, both the non-denoised and DL denoise images (**D**) appear equally pixelated, and there are minimal changes in tractography. The tensor ellipsoid representation of intravoxel eigen vectors is substantially larger and more numerous in the 2 mm^3^ voxel size (**B**,**C**) compared to the 2 mm × 2 mm × 3 mm voxel size (**E**,**F**).

Twenty-seven subjects (14 girls, 13 boys) were included in the study; each subject had 2 DTI sequences to which a DL reconstruction algorithm was applied (*n* = 27 subjects, *n* = 54 non-denoised DTI scans, *n* = 54 DL reconstructed scans). In all subjects, isovolumetric 2 mm^3^ DTI scans exhibited a visibly higher quantity of tensor ellipsoids compared to the 2 mm × 2 mm × 3 mm acquisition, a more defined diffusion direction was observed in the smaller voxel size, as shown in Figure 1. Fiber tract count, volume, and length were consistently larger in both the femur and tibia physes when using the isovolumetric 2 mm^3^ voxel size in both the DL reconstructed scans and non-denoised scans, as shown in Table 1 and Table 2. 

**Figure 2:**
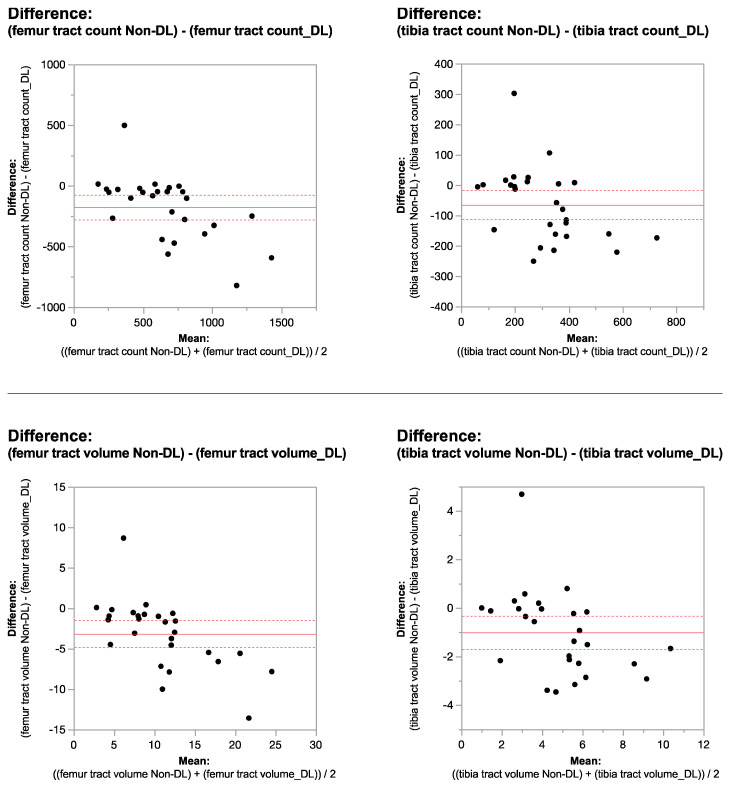
Bland–Altman plots regarding the difference in DTI metrics for same physeal ROIs between DL reconstructed-and non-denoised (Non-DL) image DTIs. The horizontal axis represents the mean of the two methods and the vertical axis, the difference between them. The solid line (red) shows the mean difference (close to zero) and the dashed lines show the 95% limits of agreement.

**Figure 3:**
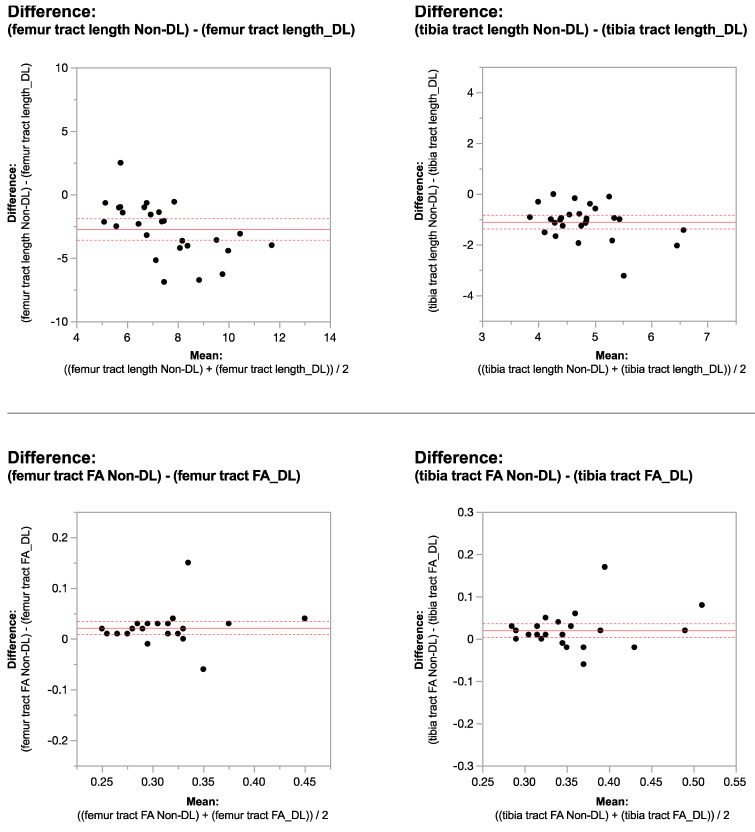
Bland–Altman plots regarding the difference in DTI metrics for same physeal ROIs between DL-reconstructed and non-denoised (non-DL) image DTIs. The horizontal axis represents the mean of the two methods and the vertical axis, the difference between them. The solid line (red) shows the mean difference (close to zero) and the dashed lines show the 95% limits of agreement.

Applying the reconstruction algorithm led to an increase in femorotibial tract count, volume, and length in both DL reconstructed voxel dimensions compared to non-denoised scans (Table 3 and Table 4). DTI metrics showed a greater increase in scans acquired using isovolumetric 2 mm^3^ compared to the scans acquired with 2 mm × 2 mm × 3 mm voxel dimensions (*p* = 0.04). Diffusion metrics (tract count, volume, and length) derived from the DL reconstructed scans were significantly higher from the non-denoised scan DTI metrics in both the femur and tibial physes using the 2 mm^3^ voxel dimension (*p* < 0.001 (Table 3, Figure 2 and Figure 3). 

**Figure 4:**
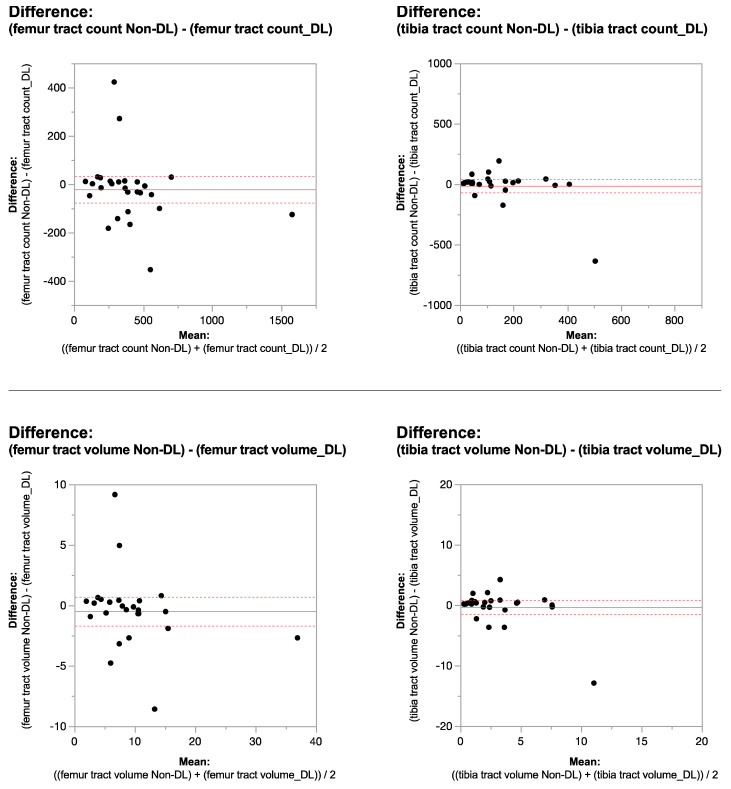
Bland–Altman plots regarding the difference in DTI metrics for same physeal ROIs between DL reconstructed and non-denoised (non-DL) DTIs. The zero value is indicated by the red line. The horizontal axis represents the mean of the two methods and the vertical axis, the difference between them. The solid line (red) shows the mean difference (close to zero) and the dashed lines show the 95% limits of agreement. The mean is very close to zero for most cases, indicating little difference between the methods, and the range of LoA is relatively small indicating a good numerical agreement in the methods among the majority of patients for the 2 mm × 2 mm × 3 mm voxel size.

**Figure 5:**
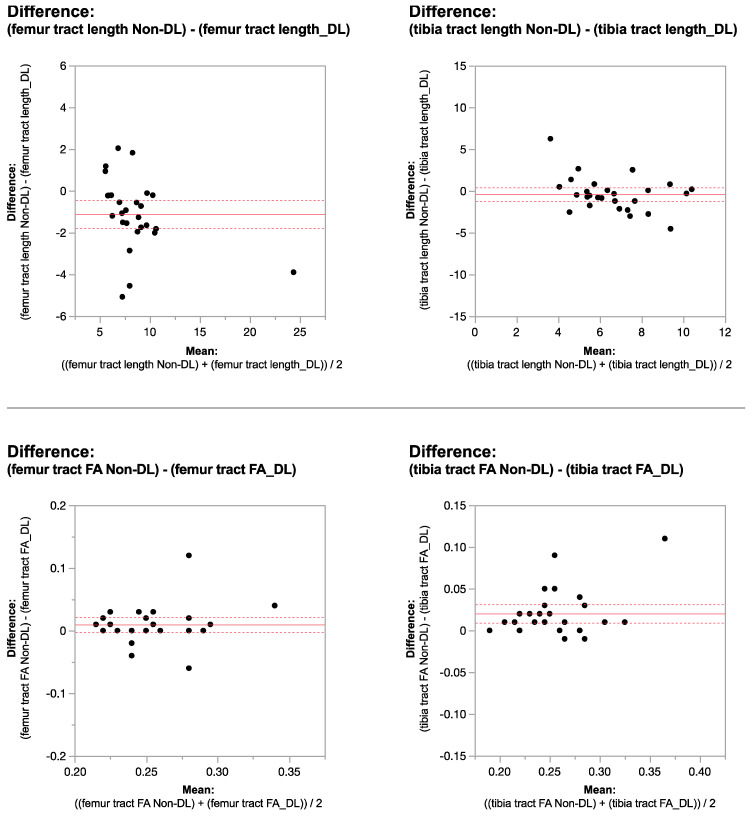
Bland–Altman plots regarding the difference in DTI metrics for same physeal ROIs between DL reconstructed and non-denoised (non-DL) DTIs. The zero value is indicated by the red line. The horizontal axis represents the mean of the two methods and the vertical axis, the difference between them. The solid line (red) shows the mean difference (close to zero) and the dashed lines show the 95% limits of agreement. The mean is very close to zero for most cases, indicating little difference between the methods, and the range of LoA is relatively small indicating a good numerical agreement in the methods among the majority of patients for the 2 mm × 2 mm × 3 mm voxel size.

**Figure 6:**
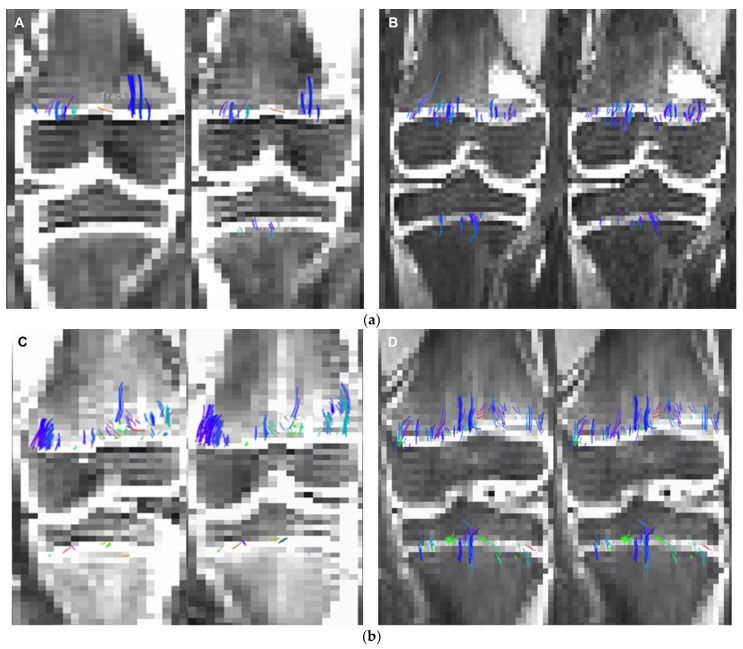
(**a**). 2 mm × 2 mm × 3 mm (**A**) non-denoised (**left**) and 2 mm × 2 mm × 3 mm denoised (**right**) versus 2 mm^3^ (**B**) non-denoised (**left**) and denoised (**right**) in a 9-year-old girl. There is an evident increase in fiber tract on the denoised images acquired with a 2 mm^3^, while denoised and non-denoised images are very similar using 2 mm × 2 mm × 3 mm voxel size. (**b**). 2 mm × 2 mm × 3 mm (**C**) non-denoised (**left**) and denoised (**right**), versus 2 mm^3^ (**D**) non-denoised (**left**) and denoised (**right**) in a10 year-old boy. There is an evident increase in fiber tract on the denoised images acquired with a 2 mm^3^, while denoised and non-denoised images are very similar using 2 mm × 2 mm × 3 mm voxel size.

When comparing DL versus non-denoised diffusion metrics in femur and tibia using the 2 mm × 2 mm × 3 mm voxel dimension there were no significant differences between tract count (*p* = 0.1, *p* = 0.14) tract volume (*p* = 0.14, *p* = 0.29), or tibial tract length (*p* = 0.16); femur tract length exhibited a significant difference (*p* < 0.01) (Table 4, Figure 4 and Figure 5). DL reconstruction resulted in a significant decrease in femorotibial fractional anisotropy (FA) for both voxel dimensions (*p* < 0.01) (Figure 3 and Figure 5). Figure 6A,B show DTI tractography changes in non-denoised and denoised 2 mm^3^ versus non-denoised and denoised 2 mm × 2 mm × 3 mm in a 9-year-old girl and a 10-year-old boy.

SNR values were significantly higher in the non-denoised femur and tibia ROIs in the 2 mm × 2 mm × 3 mm voxel dimension compared to the 2 mm^3^ voxel size (*p* < 0.0001), a pattern observed both before and after applying DL-denoising (Table 5). Following the application of DL-denoising, the femur and tibia ROI SNR on b0 exhibited a 39% and 41% increase in the 2 mm3 voxel dimension, respectively, in contrast to the 37% and 38% increase in the 2 mm × 2 mm × 3 mm (Table 5). Moreover, the SNR for the femur and tibia ROI on b600 experienced a 39% and 40% increase in the 2 mm^3^ voxel size, whereas a more pronounced increment of 40% and 42% was observed in the 2 mm × 2 mm × 3 mm (Table 6).

## 4. Discussion

Voxel dimension is one of the factors that influences fiber tracking and the degree of PVEs [25]. Larger voxel sizes can contain more than one dominant diffusion orientation, thereby causing possible errors in estimating the primary tensor direction which ultimately impacts fiber tracking and the resultant diffusion metrics [1,25]. This may explain the markedly smaller tensor ellipsoid representations inside a voxel with a less defined direction observed in the larger voxel dimension (which results in lower microscopic resolution) compared to the smaller isotropic voxel size used (2 mm^3^). The relationship between voxel resolution and image quality is evident in Figure 1, where the knee bones and physes are more sharply defined on the isotropic 2 mm^3^ voxel size [31].

The use of larger voxels resulted in smaller fiber tract diffusion metrics. Larger 3D voxels can cover the entire field of view (FOV) and thickness with fewer voxels overall at a lower spatial resolution, the opposite is true when using smaller voxel sizes, hence fewer tensors overall are calculated on bigger voxels (as more area is covered by one 3D voxel and a single dominant tensor is calculated per voxel) accounting for lower tract count, length, and volume when using a larger voxel size. 

We hypothesize the significant increase in both femur and tibia fiber tract count, volume, and length after denoising isovolumetric 2 mm^3^ scans is due to the removal of intrinsically increased noise by the applied reconstruction algorithm. Diffusion metrics on the bigger voxel size in same subject scans, however, had better SNR and lower microscopic resolution, which was not improved with the reconstruction algorithm. This may possibly explain the small changes in tract count and volume in both physis after reconstruction algorithm application, which were not significant. The change in femoral tract length after denoising the 2 mm × 2 mm × 3 mm voxel size was small yet statistically significant, suggesting tract length is more sensitive to small SNR changes when compared to tract volume and tract count. In previous femur and tibia physeal DTI studies evaluating growth, tract length results have been variable: showing poor interobserver reliability compared to other fiber tract diffusion metrics (count, volume, length, and FA) evaluated in the same specimens [8], and it also did not show the expected change with age in animal models [8], which could suggest tract length is susceptible to small changes. 

FA is the measurement of the degree of restricted water diffusion, calculated from the eigen value of the diffusion tensor [32]. In brain white matter, FA has been seen to decrease steadily after 20 years of age. Previous studies on the knee physes, have shown increasing FA with age as the closing physes, now ossified cartilage, show greater water diffusion restriction [7,9]. FA contrasts the principal eigenvalues of diffusivity and is considered to be limited by noise, making it susceptible to voxel size effects [33]. A previous study evaluating FA in brain white matter fiber tracts in different subjects using increasing voxel sizes found voxel size to significantly affect FA with smaller voxels giving higher FA values and reporting the impact was strongest at the highest spatial resolutions [33]. These mirrors our findings where same subject mean femur and the tibia FA values were 0.34 and 0.36 (2 mm^3^) and 0.26 for both femur and tibia (2 mm × 2 mm × 3 mm). 

High noise levels can bias DTI measurements, which can consequently produce errors in estimation of fiber tract parameters [34]. Low SNR can cause overestimation and underestimation of the largest and smallest eigenvalues, respectively [35]. A previous study evaluating DL noise reduction effects on FA in CNS structures in 20 patients, performed one image acquisition (NAQ1) versus five image acquisitions (NAQ5), and compared FA values after DL denoising was applied in NAQ1 [36]. They found FA to be overestimated when the number of image acquisitions was one (NAQ1), and after denoising NAQ1′s FA decreased and came closer to that of NAQ5 [36]. In our study, a similar decrease in FA values on both spatial resolutions after application of the DL reconstruction occurred likely due to noise elimination and the resultant increased signal, with greater signal achieved in the intrinsically noisier 2 mm^3^ spatial resolution explaining the greater drop in FA in the smaller voxel size (non-denoised versus denoised FA for the femur, 0.31 and 0.29, and the tibia, 0.36 to 0.34). This finding is also consistent with previous studies that low SNR leads to overestimation of FA on skeletal muscle [37], and the positive bias in FA values on peripheral nerve was removed after denoising [18].

We observed that the ROI SNR values for both the femur and tibia were higher when utilizing the larger voxel size (2 mm × 2 mm × 3 mm), irrespective of denoising. This aligns with the acknowledged trade-off between spatial resolution and SNR. Initially we anticipated a higher increase in SNR after denoising the smaller voxel dimensions (2 mm^3^). The signal-to-noise ratio (SNR) quantitatively increased slightly more for the b600 images in the 2 mm × 2 mm × 3 mm voxel size than for the 2 mm^3^. The 2 mm × 2 mm × 3 mm demonstrated higher SNR before denoising and the subsequent increase in SNR did not impact the metrics as observed in the smaller, noisier voxel dimensions. 

The children imaged in this study are part of an ongoing longitudinal cohort, focusing on predicting growth in healthy children using DTI of the physeal–metaphyseal complex. As a standard practice, the left knee was chosen for imaging as it was more likely to be the non-dominant knee and less prone to injury, ensuring consistency and reducing potential confounding factors for analysis. No differences are predicted in the equally healthy right contralateral extremity in terms of SNR or imaging acquisition that would alter the findings on this study. 

This study is limited by the small sample size used. To address this, the methods could be replicated in a bigger subject population to determine if the effects observed are consistent. The data can also be compared in groups of children in the clinical setting, with musculoskeletal disease for example and/or adult population. This study provides information to support the leveraging of denoising algorithms, such as AIRTM Recon DL, on DTI acquisition with smaller voxel volumes. The noise is reduced while preserving the higher spatial resolution, allowing for more accurate quantification of diffusion metrics. 

This approach could address the drawbacks of lower SNR associated with smaller voxel volumes and capitalizes on their better spatial resolutions. This allows clinicians a clearer view of growth plate tissue microstructures without sacrificing signal. In addition, it may be possible to take advantage of better image resolution without a greater acquisition time which is essential when imaging pediatric subjects. In cases where there will be various same subject acquisitions, FA values are more reliable when they are denoised than when they are not. 

Recognizing DTI’s inherent sensitivity to noise, which significantly impacts the reliability of metrics such as fractional anisotropy (FA) and tract count, our discussion delves into the effectiveness of the DL denoising algorithm. This pretrained prototype is adept at generating noise-tolerant, reliable DTI metrics, a critical advancement for interpreting complex microstructures like the knee growth plate in the context of skeletal growth. This capability to reduce noise while preserving essential diffusion signals is pivotal, especially for the knee growth plate, where precise visualization and accurate tract counts and other diffusion metrics are crucial for assessing prediction of post-imaging growth and growth plate closure as well as positive and negative effect of treatments like growth hormone administration for potential growth disorders in pediatric patients. The significant improvements in DTI metrics following denoising underscore the impact of DL techniques in addressing DTI’s traditional challenges. Given its fixed pretrained parameters, this inference network seamlessly translates across different body parts for DTI scans, negating the need for retraining. This characteristic is helpful in clinical and research settings, offering a stable, reliable tool to enhance DTI data quality.

## 5. Conclusions

A DL reconstruction algorithm may lead to significant increase in femorotibial DTI metrics (tract count, volume, and length) when applied on smaller voxel sizes, while causing a significant decrease in FA regardless of voxel dimension, larger cohorts should be used to asses validation. 

Leveraging denoising algorithms could address the drawbacks of lower SNR associated with smaller voxel volumes and capitalizes on their better spatial resolutions, allowing for more accurate quantification of diffusion metrics.

## Figures and Tables

**Table 1:** Denoised AIr Recon DL DTI metrics: 2 mm^3^ versus 2 mm × 2 mm × 3 mm.

DTI METRIC	AIr Recon DL	AIr Recon DL	*p*-Value
	Isovolumetric 2 mm^3^	2 mm *×* 2 mm *×* 3 mm	
femur tract count	753.03 ± 409.17	410.37 ± 308.29	<0.0001 *
femur tract volume	12.47 ± 7.05	9.52 ± 7.17	<0.0006 *
femur tract length	8.83 ± 2.48	9.07 ± 3.93	0.9
femur FA	0.29 ± 0.04	0.25 ± 0.02	<0.0001 *
tibia tract count	341.62 ± 187.3	137.44 ± 177.31	<0.0001 *
tibia tract volume	5.34 ± 2.7	4.50 ± 8.36	0.0005 *
tibia tract length	5.36 ± 0.84	6.81 ± 2.42	0.002 *
tibia FA	0.34 ± 0.05	0.24 ± 0.03	<0.0001 *

Wilcoxon signed rank test; *p*-value < 0.05 was considered significant (*).

**Table 2:** Raw Data DTI metrics: 2 mm^3^ versus 2 mm × 2 mm × 3 mm.

DTI METRIC	Raw Data	Raw Data	*p*-Value
	Isovolumetric 2 mm^3^	2 mm × 2 mm × 3 mm	
femur tract count	576.85 ± 257.21	388.62 ± 274.57	<0.0001 *
femur tract volume	9.3 ± 4.45	9.03 ± 6.4	0.13
femur tract length	6.11 ± 1.39	7.96 ± 3.25	0.001 *
femur FA	0.31 ± 0.04	0.26 ± 0.03	0.0001 *
tibia tract count	277.22 ± 133.89	123.44 ± 112.03	0.0001 *
tibia tract volume	4.33 ± 1.99	2.74 ± 2.2	0.0001 *
tibia tract length	4.26 ± 0.62	6.43 ± 1.75	0.0001 *
tibia FA	0.36 ± 0.06	0.26 ± 0.04	0.0001 *

Wilcoxon signed rank test; *p*-value < 0.05 was considered significant (*).

**Table 3:** Raw data versus DL denoised DTI metrics: 2 mm^3^.

DTI METRIC	Raw Data	AIr Recon DL	*p*-Value
	Isovolumetric 2 mm^3^	Isovolumetric 2 mm^3^	
femur tract count	576.85	753.03	<0.0001 *
femur tract volume	9.3	12.47	<0.0001 *
femur tract length	6.11	8.83	<0.0001 *
femur FA	0.31	0.29	<0.0001 *
tibia tract count	277.22	341.62	0.013 *
tibia tract volume	4.33	5.34	0.001 *
tibia tract length	4.26	5.36	<0.0001 *
tibia FA	0.36	0.34	0.005 *

Wilcoxon signed rank test; *p*-value < 0.05 was considered significant (*).

**Table 4:** Raw data versus DL denoised DTI metrics: 2 mm × 2 mm × 3 mm.

DTI METRIC	Raw Data	AIr Recon DL	*p*-Value
	2 mm × 2 mm × 3 mm	2 mm × 2 mm × 3 mm	
femur tract count	388.62	410.37	0.1
femur tract volume	9.03	9.52	0.14
femur tract length	7.96	9.07	0.001 *
femur FA	0.26	0.25	0.017 *
tibia tract count	123.44	137.44	0.14
tibia tract volume	2.74	4.5	0.29
tibia tract length	6.43	6.81	0.16
tibia FA	0.26	0.24	<0.0001 *

Wilcoxon signed rank test; *p*-value < 0.05 was considered significant (*).

**Table 5:** SNR values in the non-denoised femur and tibia ROIs: 2 mm × 2 mm × 3 mm voxel dimension compared to 2 mm^3^ voxel size.

Voxel Size Comparison of ROI SNRs for b0 and b600 DTI with and without DL			
2 mm^3^	DL	Non-DL	*p*-value
femur_b0	44.2	31.7	<0.0001 *
femur_b600	18.9	13.7	<0.0001 *
tibia_b0	36.4	25.8	<0.0001 *
tibia_b600	16.6	11.9	<0.0001 *
**2 mm × 2 mm × 3 mm**	**DL**	**Non-DL**	***p*** **-value**
femur_b0	67.1	49.0	<0.0001 *
femur_b600	29.6	20.9	<0.0001 *
tibia_b0	54.1	39.3	<0.0001 *
tibia_b600	25.4	17.9	<0.0001 *

Wilcoxon signed rank test; *p*-value < 0.05 was considered significant (*).

**Table 6:** SNR Increase.

SNR Increase (Mean Difference SNR/Non-DL SNR)	2 mm^3^	2 mm × 2 mm × 3 mm
femur_b0	0.39	0.37
tibia_b0	0.41	0.38
femur_b600	0.39	0.42
tibia_b600	0.4	0.42

## Data Availability

Data generated and analyzed during this study are not publicly available due to subject imaging containing protected health information but are available from the corresponding author on reasonable request.
